# Ensuring that psychological interventions are delivered as intended on mental health inpatient wards

**DOI:** 10.1111/bjc.12510

**Published:** 2024-10-28

**Authors:** Katherine Berry, Fritz Handerer, Sandra Bucci, Georgia Penn, Helen Morley, Jessica Raphael, Karina Lovell, Owen Price, Dawn Edge, Richard J. Drake, Gillian Haddock

**Affiliations:** ^1^ Division of Psychology and Mental Health, School of Health Sciences, Faculty of Biology, Medicine and Health, Manchester Academic Health Sciences The University of Manchester Manchester UK; ^2^ Greater Manchester Mental Health NHS Foundation Trust Manchester UK; ^3^ Division of Nursing, Midwifery and Social Work, School of Health Sciences, Faculty of Biology, Medicine and Health the University of Manchester Manchester UK

**Keywords:** case formulation, fidelity, implementation, inpatient treatment, psychosis, schizophrenia, therapeutic alliance, therapeutic factors

## Abstract

**Objectives:**

Talk, Understand and Listen for InPatient Settings (TULIPS) was a multi‐centred randomized control trial of an intervention that aimed to increase patient access to psychological therapies on acute mental health wards. This paper aims to: (i) describe a strategy for designing a psychological intervention that is implementable in inpatient mental health settings; (ii) describe methods for assessing the fidelity of interventions within these settings; (iii) report on the extent to which fidelity was achieved in the TULIPS trial.

**Methods:**

The TULIPS intervention was designed using information from a systematic review, stakeholder interviews, pilot work and a consensus workshop. We assessed fidelity to the model in terms of the delivery and dose of essential elements of the intervention, quality of intervention delivery, engagement of participants with the intervention and differentiation between the intervention and usual care.

**Results:**

Although the TULIPS intervention targeted known barriers to the delivery of psychological interventions on mental health wards, we found issues in implementing aspects of the intervention that were dependent upon the participation of members of the multidisciplinary team. Psychologists were able to overcome barriers to delivering individual therapy to patients as this provision was not reliant on the availability of other staff.

**Conclusions:**

The intervention period in the study was 6 months. A greater period of time with a critical mass of psychological practitioners is needed to embed psychological interventions on inpatient wards. Our fidelity framework and assessment methods can be used by other researchers implementing and testing psychological therapies within inpatient environments.

## INTRODUCTION

Care on acute mental health wards is often criticized for its excessive costs, focus on biomedical approaches and a lack of person‐centred engagement (Staniszewska et al., 2019). Contrary to the current practice, most mental health inpatients want to have the option to take up talking therapy, and recent National Health Service (NHS) guidelines demand that acute wards should be therapeutic settings offering psychological interventions (NHS England, 2023). Although there is evidence that psychological interventions can positively affect functioning and reduce readmission rates (Wood et al., 2020), most literature reviews conclude that the rigour and quality of the conducted studies in this field is insufficient to draw any conclusions (Barnicot et al., 2020; Jacobsen et al., 2018; Paterson et al., 2018).

One of the most commonly identified problems with the studies evaluating psychological interventions within acute mental health settings is that the studies report either low fidelity rates or use no fidelity measures at all (Evlat et al., 2021; Jacobsen et al., 2018). Intervention fidelity is broadly defined as the extent to which an intervention is being implemented as intended. High fidelity on the one hand ensures internal validity as it keeps the independent variable (the intervention) consistent, and on the other hand ensures external validity as it demonstrates that an intervention is implementable within different contexts (Ginsburg et al., 2021). If an intervention is delivered with low fidelity, valid inferences cannot be made as any present or absent effects might either be due to the intervention or deviation from the protocol.

There are two main reasons for the low fidelity rates and/or poor fidelity reporting of psychological interventions in acute wards. First, psychological interventions are typically complex interventions, consisting of multiple interacting components, delivered by different individuals. This complexity is more extreme in the inpatient environment where multiple different staff are delivering and influencing care for one individual at any one time and there is a need to vary interventions depending on the patient's fluctuating needs and levels of distress. For example, a recent systematic review of the implementation of psychological therapies on acute inpatient wards, largely consisting of cognitive behavioural therapy interventions, identified that key barriers were the busy nature of the ward, ward staff not being suitably train and the acute nature of patients' mental health (Evlat et al., 2021). Second, there are multiple dimensions of fidelity which are not consistently defined (Toomey et al., 2020) and most study reports fail to explicate their conceptualisation of fidelity (Slaughter et al., 2015).

In order to interpret the findings of randomized controlled trials of complex interventions, it is essential to understand fidelity and other factors related to implementation (Moore et al., 2015; Skivington et al., 2021). For example, it will only be possible to decide if and which form of psychological therapy is most helpful in inpatient settings when interventions are being implemented and evaluated with high fidelity. We therefore set out to develop a psychological intervention that could be implemented with high fidelity in acute inpatient mental health wards. The Talk, Understand and Listen for InPatient Settings (TULIPS) intervention (described more fully in the method) is a ward‐based intervention delivered by clinical psychologists that aims to increase patient access to psychological therapy on acute mental health ward through team formulation and therapy carried out by both psychologists and nursing staff. The TULIPS intervention was tested in a single blind cluster randomized controlled trial comparing 17 acute mental health wards receiving the TULIPS intervention plus usual care with 17 wards receiving usual care alone Berry, Raphael, Wilson, et al., (2022). In this paper, we focus on the fidelity of the intervention. We had three aims, to: (i) describe a strategy for designing a complex psychological intervention that aims to be implementable with high fidelity in acute mental health settings by addressing identified barriers and facilitators; (ii) outline a framework and associated set of measures to comprehensively assess the multiple dimensions of fidelity relevant to implementing psychological intervention within acute mental health settings; (iii) report the fidelity rates of the TULIPS intervention, tested in 17 acute mental health wards across the UK.

## METHODS

### The strategy for designing the TULIPS intervention

We developed the TULIPS intervention in four steps. This process was guided by Medical Research Council guidance for the development of complex interventions and drew on information from a range of different sources to aid implementation of the intervention (Moore et al., 2015; Skivington et al., 2021). Initially, we conducted a meta‐synthesis review of the literature to identify barriers and facilitators of implementing psychological interventions in acute mental health wards (Study 1: Raphael, Hutchinson, et al., 2021). Next, we conducted semi‐structured interviews with 26 staff members, 22 patients, and 12 carers, to explore their perspectives on barriers to delivering and accessing psychological therapies in inpatient settings (Study 2: Berry, Raphael, Haddock, et al., 2022). The findings of the review and the interviews together informed the initial TULIPS intervention protocol. To finalize this protocol and address unanswered questions, we conducted a consensus conference with more than 20 nationally drawn experts in the field, including patient and carer representatives, mental health service providers and researchers (Study 3: Raphael et al., 2020). Recommendations arising from this conference were intended to ensure the intervention could be realistically implemented on acute wards. The resulting TULIPS intervention was successfully deployed in two wards and evaluated in a feasibility study (Study 4: Raphael, Price, et al., 2021). Table 1 summarizes the findings from each of these four studies and shows how findings from these individual studies influenced the TULIPS intervention design.

**TABLE 1:** Identified facilitators and barriers from each study and influence on the design of the TULIPS intervention.

No.	Study	Identified barriers and facilitator	Influence on TULIPS intervention design
1.	Overcoming barriers to implementing ward‐based psychosocial interventions in acute inpatient mental health settings: A meta‐synthesis (Raphael, Price, et al., 2021)	Barrier: Staff and patients lack knowledge of psychological interventions Staff lack confidence responding to patients' emotional distress Leadership is ambiguous regarding psychological interventions Nurse's work compartmentalized into a series of tasks Facilitator: The need to increase the psychological‐mindedness of the whole team to support the work of more experienced therapist	Provide training to all ward staff to foster psychological knowledge and confidence in addressing emotional distress Include nurses in the provision of psychological interventions Include all ward staff in formulation development to develop a more psychologically minded ward culture Provide a selection of structured workbooks focused of mental health issues for nurses to complete with patients
2.	Exploring how to improve access to psychological therapies on acute mental health wards from the perspectives of patients, families and mental health staff: qualitative study (Berry, Raphael, Haddock, et al., 2022)	Barriers: Psychologists not being physically present on the wards Staff burnout due to overwhelming workloads and traumatic work experiences Facilitator: As the main function of inpatient care is emotional containment, psychological therapy should not open up issues that cannot be addressed within a short stay hospital admission	Base clinical psychologists on the ward to increase their physical presence and accessibility Provide group sessions to staff members on wellbeing and reflective practice to address burnout and compassion fatigue Individual therapy sessions with psychologists and patients focused on factors that prevent discharge and contribute to readmission
3.	Conducting a consensus conference to design a psychology service model for acute mental health wards (Raphael et al., 2020)	Barrier Nursing staff lacking confidence in delivering psychological interventions Facilitators: Flexibility with respect to the therapeutic model used in formulation and therapy sessions Prioritizing patients for formulation or therapy who have longest admission and most frequent readmissions Provide regular reflective practice sessions	Psychologists to provide fortnightly supervision to nurses in the delivery of psychological interventions Allowing psychologists to formulate and deliver psychological therapy informed by different psychological models Formulation and therapy work prioritize patients with longest admissions and most frequent readmissions
4.	A study on the feasibility of delivering a psychologically informed ward‐based intervention on an acute mental health ward (Raphael, Price, et al., 2021)	Barrier: Staff not being available to deliver interventions or attend sessions with the psychologists Facilitator Flexible delivery of the intervention with prioritization of different components depending upon the ward's individual circumstances Regular supervision of clinical psychologists focused on issues of intervention delivery	Increased focus on obtaining ward manager buy in to the intervention so staff time can be allocated to intervention delivery Regular monitoring of intervention delivery Flexibility regarding which components of the intervention are prioritized Psychologists have regular access to supervisions with senior staff experienced in inpatient working to reflect on and problem solve barriers to delivery

### TULIPS intervention

The intervention comprises three levels and was designed to be delivered by Health Care Profession Council registered clinical psychologists. The three levels constituted a stepped model of care whereby level 1 was delivered to all patients on the ward and patients were stepped up level 2 or level 3 depending on need. Psychological formulations were used to determine level of need. Level 1 involves introductory training to ward staff on trauma‐informed mental health care, case formulation and self‐compassion. Following the training, clinical psychologists develop a formulation of each patient's needs either in collaboration with ward staff or together with a patient's‐named nurse. In addition, psychologists facilitate group sessions for ward staff on wellbeing, reflective practice and attend multidisciplinary team meetings. In level 2, psychologists train and supervise a smaller number of staff on the ward team in delivering structured, brief intervention topics selected in collaboration with the ward manager. Topics include self‐harm, psychosis, emotional regulation, low mood, anxiety, anger and low self‐esteem. In level 3, psychologists deliver up to 16 individual therapy sessions to in‐patients with particularly complex needs. The sessions focus on factors keeping the patient on the wards or contributing to readmissions.

### Framework for assessing fidelity

In conceptualizing aspects of fidelity that were pertinent to this study, we were guided by previous literature on assessing fidelity in complex interventions (Bellg et al., 2004; Dusenbury et al., 2003; Ginsburg et al., 2021; Walton et al., 2020). We focused on four dimensions of fidelity: (1) the delivery and dose of essential elements of the intervention; (2) quality of intervention delivery; (3) engagement (to what degree staff and patients engage with the intervention); (4) differentiation (the degree to which it is possible to differentiate between elements of the intervention and usual care).

We present the measures of fidelity used in this study in accordance with the fidelity dimension they assess. The dimensions are not mutually exclusive, and some measures are relevant for the assessment of more than one dimension. Table 2 summarizes the fidelity measure and its respective dimensions, along with any targets we aimed to meet.

**TABLE 2:** Summary of fidelity measures and their respective dimensions.

No.	Fidelity dimension	Measure	Any associated targets
1	Delivery and dosage	Training log (which training provided, attended by how many staff members) Log of provided therapy sessions (number of patients who received therapy and number who declined) Psychologists' diaries detailing formulations (number undertaken; in group or with named nurse; duration), staff group sessions (number of reflective practice sessions; staff wellbeing; incidents review took place), supervision to staff, patient therapy sessions (number of sessions), MDT sessions attended (number and duration) and own supervision (duration) Record of number of level 2 interventions (group and individual) delivered by ward staff including topic and number of patients attending groups	Delivering a half day training on mental health and trauma to all permanent staff and optional further training of up to 4 half days in specific intervention 3 h of formulations (either in team or psychologist with named nurse) per week covering at least 2 patients per week 3 h staff supervision per week 1 h of staff group sessions per week 2 h therapy sessions per week 3 h MDT meetings per week 1 h of own supervision per week Nurses delivering level 2 individual and group sessions (exact number not specified)
2	Quality	Training evaluation (helpfulness and quality of training, areas for improvements) Team Formulation Quality Scale (TFQS): 15 items for independent rater/ 30 items for self‐report to evaluate quality of recorded team formulation sessions Working Alliance Inventory (shortened versions) to measure quality of relationship between therapist and patient Cognitive Therapy Scale for Psychosis (CTS‐Psy) 30 items to assess quality of recorded individual therapy sessions Evaluation of helpfulness of received supervision	A training evaluation with an average score of more than 3 (which leans towards helpful/ positive) Team Formulation Quality Scale score of at least 20/30 based on data from study used to develop the measure Average of WAI self‐report score of more than 3.5 which leans towards more positive alliance A CTS‐Psy score of 15 or above accounting for us only using a proportion of the measure A helpfulness of supervision scale with an average score of more than 3 (which leans towards helpful/ positive)
3	Engagement	A measure to assess engagement of staff members with intervention rated by psychologists Cancellation of meetings/ training sessions (MDT sessions; supervision) Number of offers of therapy declined and cancelled therapy sessions	Explorative analysis of the generated questions regarding staff engagement Explorative analysis of cancellation of other meetings/training due to lack of comparable data Up to 20% non‐attendance at therapy session based on previous research
4	Differentiation	Log of staff movement to monitor any contamination of staff members moving from intervention to control wards and vice vers. Log of psychological input on wards as part of usual care	No targets set due to the pragmatic nature of the TULIPS trial

#### Delivery and dosage

We defined core elements of the TULIPS intervention in line with the three levels of intervention outlined above: (i) developing a formulation for every patient either together with the whole ward team or together with a patient's individually‐named nurse; (ii) facilitating psychological skills of the staff team by training and supervising ward staff in delivering brief structured interventions to patients; (iii) psychologists providing individual therapy to patients with more complex needs. The psychologists' work at the 3 levels was supported through their attendance at MDT meetings, their delivery of staff reflective practice and/or staff wellbeing sessions, and through receiving their own supervision. Dosage refers to how often intervention elements are delivered; we collected this information alongside information about delivery due to overlap between the constructs.

We measured delivery and dosage in several different ways, including a training log to capture the number and type of training sessions delivered, a log of patients who were offered and declined individual therapy, and electronic or paper records of groups or therapy sessions delivered by ward staff. Psychologists also completed a daily electronic diary, which included information on the number of formulations developed, the length of time spent engaged in formulation, the number of staff reflective practice or well‐being sessions delivered, supervision sessions provided to staff, therapy sessions provided to patients, MDT sessions attended, and peer or personal supervision sessions received. We appraised the dosage of intervention elements against the number of hours that psychologists were recommended to spend on each activity per week in the TULIPS manual (see Table 2 for recommended hours for each activity).

#### Quality of intervention delivery

The second dimension of fidelity we considered was the quality of intervention delivery.

To assess the quality of the training provided, staff members attending the training completed an evaluation form that included three questions (usefulness of provided information, quality of presentation, usefulness for clinical practice) each scored on a 5‐point Likert scale, and three open questions pertaining to aspects of the training that were most and least helpful and areas for improvements.

To assess the quality of team formulation intervention delivery, the Team Formulation Quality Scale (TFQS; Bucci et al., 2021) was applied to all available audio recorded team formulation sessions (*n* = 46). The TFQS consists of 15 items, scored as either *yes, to some extent, or no*, that fall into the two sections: *structure* and *content*. Ratings of quality were made by a trainee clinical psychologist and a consultant clinical psychologist experienced in team formulation, both independent from the study team. We assessed whether the raters were reliable with each other and 20% of the available recordings were also evaluated by author KB to provide a further reliability check. In addition, to the independent ratings of team formulation, psychologists delivering the intervention completed a self‐report version of the TFQS scale also scored out of 30. We were interested in the extent to which psychologist's own perception of formulation quality corresponded with independent assessments of quality and wanted to capture information about the quality for sessions that were not audio recorded primarily due to lack of staff consent.

The quality of therapy sessions carried out by psychologists was assessed by measuring the working alliance between psychologists and patients using the short version of the Working Alliance Inventory (Hatcher & Gillaspy, 2006), completed after sessions 3, 6, 9, 12 and 15. The WAI short version consists of 12 items for patients and 10 items for therapists, both rated on a 5‐point Likert scale. Working alliance was assessed for all patients who received individual therapy with a psychologist and who gave written informed consent to complete the measures.

The quality of 20% of the audio recorded therapy sessions (*n* = 15) was also assessed by two independent psychological therapists using components of the Cognitive Therapy Scale for Psychosis (CTS‐Psy). We only focused on the general items of the CTS‐Psy and omitted the second section of the original measure that focuses on the assessment of CBT‐specific skills, as we allowed for different therapeutic models to be applied. The included 30 items are equally distributed across the five dimensions: agenda, feedback, understanding, interpersonal effectiveness, collaboration. Each item is scored as either present or absent. The scale has been shown to be a valid indicator of therapy skills (Haddock et al., 2001). A proportion of all rated audio‐recordings (35%) was evaluated independently by both of the raters and author KB to test for reliability (*n* = 5).

Finally, the quality of the therapist's own supervision sessions was assessed by a 5‐point Likert scale created for the purposes of the study to assess perceived helpfulness.

#### Engagement

An intervention not only has to be delivered according to the essential elements of the protocol, but also the people receiving the intervention must engage with the intervention. In this study, we assessed how well patients and ward staff engaged with the TULIPS intervention. To measure staff engagement, we developed a short questionnaire which psychologists completed in relation to each staff member on the ward who had given consent to take part in the TULIPS trial. We did not collect data about staff who had not consented to be part of the trial even though these individuals were exposed to the intervention by virtue of the cluster design and the fact it was a ward‐based intervention. The questionnaire consisted of seven questions rated on a 4‐Point Likert scale and assessed both staff physical attendance and emotional engagement within supervision and team formulation sessions as well as engagement with the psychologist more informally on the ward. In addition to the questionnaire, we assessed the number of cancelled staff group‐ and supervision sessions at a ward level. Engagement of patients was measured by counting the number of cancelled individual and group sessions.

#### Differentiation

Differentiation refers to the degree to which the implementation of an intervention is able to differentiate between the intervention and control group. To measure differentiation in the TULIPS study, research staff noted staff movement between wards, allowing us to capture any potential contamination effects when staff members moved between intervention and control wards. Ward managers and psychologists delivering the intervention were also asked to report on the psychological provision on both intervention and control wards that was part of usual care. This included details on type of professions involved and time commitment.

## RESULTS

### Delivery and dosage

The introductory training session was delivered on all wards along with 2–5 additional individual topic training packages. The three most frequent individual training topics focused on anger, psychosis and delivering individual sessions or groups. The introductory training was attended by between 7 and 29 staff members per ward (*M* = 16 staff members). We did not record the exact number of staff members employed on each ward. However, we know that the team sizes on the 11 wards at our largest research site ranged from 24 to 34 staff members, meaning that the introductory training was likely not provided to all permanent staff members. In three wards, only a small number of staff members (<10) received training.

Wards differed markedly with respect to the number of formulations recorded in the psychologists' diaries, ranging from 0 to 21, with five wards having less than five formulations recorded over the study period. We do not know exactly how many patients were admitted per ward over the intervention period. However, the average bed occupancy rate was 20 and the average length of stay was 45 days, meaning that the number of estimated admissions far exceeds the number of formulations completed. Approximately half of the formulations were developed as team formulations, one third in sessions with named nurses, and the rest were not specified. Psychologists recorded that they spent a mean of 62 min per week carrying out formulations, significantly less time than anticipated (target = 180 min per week).

Psychologists recorded that they spent a mean of 131 minutes per week on providing supervision (range 6–255 min), (compared to the target 180 min). Nurses received most of the supervision (256 individuals across all wards) followed by occupational therapists (43). Supervision sessions were attended by 1–4 staff members. Most supervision sessions provided by psychologists to staff focused on supporting staff level 2 one‐to‐one intervention work with patients, followed by staff wellbeing, reviewing groups, providing psychoeducation, ward issues and formulation.

Psychologists recorded that they spent a relatively small proportion of their time facilitating staff reflective practice or well‐being sessions. The mean time spent engaged in these activities was 21 minutes per week (range 0–42) compared to the target of 60 minutes per week. Five ward teams received none or very limited interventions of this nature. Psychologists recorded that they spent a mean of 205 minutes per week providing individual therapy sessions to patients (range 90–322 min). This represents a higher proportion of time than anticipated on providing therapy (target = 120 min per week). Across all the wards, a total of 248 patients were offered therapy with 184 patients receiving more than one session (56 declined and 8 were discharged before their first appointment was scheduled). The range of sessions offered on each ward was, however, wide, with one psychologist offering therapy to as few as four patients and another psychologist offering therapy to as many as 32 patients.

Psychologists recorded that they spent a mean of 59 min per week (range 26–93) in MDT meetings, which is lower than anticipated (target = 180 min per week). Psychologists recorded that they received a mean of 84 min of their own supervision per week (range 26–201 min), which is higher than the target of 60 min per week. Most often these supervision sessions focused on team dynamics, therapy with patients, team formulations and planning of groups.

The number of therapy sessions provided by nurses and other ward staff was limited, on average 17.2 sessions within the whole intervention period. In seven wards, ward staff provided less than four sessions.

### Quality

Staff training was rated very positively; 4.78 (.19) and 4.9 (.11) on a scale to 5 for the introductory training and the individual topic training, respectively.

The team formulation sessions had a mean score of 17.48 (SD 3.60, range 8–25) on the TFQS which ranges from 0 to 30. This mean score was lower than our pre‐specified cut off of 20.

A review of scores within each section showed that sessions lacked quality with respect to session opening (*M* = .8) and close of meeting (*M* = .71), but were notably high for collaboration (*M* = 1.84). Inter‐rater reliability across all independent raters on the TFQS was excellent (*ICC* = .926, CI 95% = .820–.971). Descriptive data for each item (scored out of 2) indicated that the psychologist scored relatively low on items addressing patient's strengths (*M* = 1.07), goals and values (*M* = .64), and social and cultural factors (*M* = 1.07), whereas psychologists scored relatively high on the life events item (*M* = 1.67). Psychologists' self‐reported the ratings on the adapted version of the TFQS were slightly higher than the independent ratings with a mean of 22.83 (SD 3.82; range 13–29). Independent and self‐ratings were moderately correlated (*r* = .333; *p* = .039).

Thirty‐six therapy dyads completed usable WAI measures at session 3. For eight dyads, we had completed measures for session 6 and three completed measures for session 9 and 12, additionally, one dyad completed the measure for session 15. As shown in Table 3, the ratings indicate very good working alliance across all timepoints and for both patients and staff.

**TABLE 3:** Quality: Working alliance inventory (SD).

WAI*	Session 3, *N* = 3 mean (SD)	Session 6, *N* = 8 mean (SD)	Session 9, *N* = 3 mean (SD)	Session 12, *N* = 3 mean (SD)	Session 15, *N* = 1 mean (SD)
Patient	4.08 (0.78)	4.55 (0.63)	4.78 (0.21)	4.64 (0.38)	4.58 (NA)
Therapist	4.07 (0.77)	4.46 (0.5)	4.63 (0.23)	4.73 (0.150)	4.8 (NA)

* Scale from 1 to 5, with higher scores reflecting better alliance.

The psychologists' therapy sessions were rated on average as 18.93 (SD 4.27, range 9–28) on the adapted version of the CTS‐Psy, which ranged from 0 to 30, reflecting an acceptable level of therapy quality. The lowest rating was 9 and the highest 28. Previous research has the criterion of 50% to determine acceptable quality (Haddock et al., 2001; Turkington et al., 2002), although our criterion was 15/30 as opposed to 30/60 given that we were not using the full scale. A review of scores within each section (scored out of 6) showed that sessions lacked quality with respect to agenda setting (*M* = 2.2) and requesting feedback (*M* = 2), but were good pertaining to understanding (*M* = 5.7), interpersonal effectiveness (*M* = 5.4), and collaboration (*M* = 4.1). The inter‐rater reliability of the raters was good (ICC = .83; 95% confidence interval .53–.98).

Finally, psychologists rated the supervision they received as very helpful, 4.64 (SD .42) out of 5.

### Engagement

In total, psychologists rated the engagement of 187 staff members using a bespoke engagement scale developed for the study. Table 4 shows the results per item. Engagement scores were slightly higher than attendance scores for both supervision and team formulation sessions, suggesting that once the barrier to attendance was surmounted, staff engaged well. Spontaneous engagement with the psychologist outside of supervision was higher than attendance of supervision, highlighting the importance of psychologist's informal accessibility.

**TABLE 4:** Staff engagement measure per item.

Item	*N*	*M* (SD)
Attendance of supervision	187	2.07 (.08)
Engagement in supervision	114	3.51 (.08)
Reflectivity in supervision	113	2.56 (.11)
Consultation outside of formal sessions	187	2.29 (.08)
Attendance of team formulation	187	2.30 (.08)
Engagement in team formulation	136	3.49 (.06)
Reflectivity in team formulation	136	2.15 (.09)

Just over a third of all types of formulation meetings (38.79%) got cancelled. The proportion of cancelled reflective practice meetings (42.11%) and staff supervision meetings (25.69%) were higher than the proportion of MDT sessions that were cancelled (4.45%). Approximately 23% of therapy sessions were cancelled. Most of the reasons for cancellation were unspecified, but 45% were reported as being due to the patient‐related factors.

### Differentiation

Of the wards allocated to the intervention arm of the trial, five had no additional psychological offer in place during the study period and 10 had <.1 WTE psychological services provided by clinical psychologists, nurse therapists or assistant psychologists. One ward employed a senior practitioner to focus on working with people with a diagnosed personality disorder .5 WTE and another ward employed an assistant psychologist at .6 WTE to work with patients. The control arm wards were very similar, with most having no or <.1 WTE psychologist provision and similarly employing a .5 WTE assistant psychologist or senior practitioner to work with people with personality disorder.

Contamination describes the risk that intervention and control groups become less differentiable due to ward staff moving across arms of a trial, making the two arms less distinct. Only six staff members moved from control to intervention wards, which poses minimal contamination risk. Only two staff members moved from intervention to control wards, which represents minimal the risk that practices from the TULIPS intervention were delivered on the control wards.

## DISCUSSION

This paper describes a strategy for designing and implementing a complex psychological intervention in inpatient mental health settings that could be delivered to high fidelity and provides a framework for assessing intervention fidelity within these settings. We also report the degree to which fidelity was achieved within the context of an evaluation of the TULIPS intervention. Reporting of fidelity for the TULIPS intervention will help to both determine the confidence with which we could make valid inferences from our trial outcome data and describe the type and extent of psychological interventions that could feasibly be delivered within inpatients settings in routine care.

The TULIPS intervention was developed based on extensive research involving stakeholder perspectives and specifically targeted known barriers to implementation of psychological interventions in acute settings. Despite this extensive and targeted approach, there were significant issues in implementing key aspects of the intervention and in particular aspects of the intervention that were dependent upon the participation of other members of the multidisciplinary team. In contrast, psychologists were able to overcome barriers to delivering individual therapy to patients in inpatient settings as this provision was not reliant on the availability of other staff. Despite concerns identified in our initial research (Berry, Raphael, Haddock, et al., 2022) about a patient's capacity to engage in therapy whilst an inpatient, both psychologists and patients reported good levels of therapeutic alliance comparable to rates reported in studies in community and inpatient settings (Shattock et al., 2018). The high alliance scores are also reflective of the skill with which psychologists were able to implement the non‐specific elements of a therapeutic relationships, with psychologists scoring high on independent ratings of session quality for collaboration, interpersonal effectiveness and understanding. The proportion of cancelled therapy sessions is comparable to other studies that implemented therapy sessions in inpatient settings (Jacobsen et al., 2020) and similar to community settings (Mitchell & Selmes, 2007), providing further confirmation that delivering therapy to inpatients is acceptable.

Similarly, when staff attended interventions delivered by the psychologists such as training, supervision and team formulation sessions, these were well received. For example, staff evaluations of the training sessions were consistently high and the psychologists rated the staff as relatively well engaged in supervision and team formulation sessions. The value of psychological input was also evidenced by the degree to which the psychologist rated staff in seeking out support and consultation outside of sessions. Previous qualitative research suggests that ward staff value psychological input and that they welcome an alternative perspective to help understand patients' needs and to support care planning (Wood et al., 2019).

The independent rating of the team formulation session was slightly lower than anticipated (18 rather than 20/30) based on the data used to develop the scale. The original data was developed using data from mental health rehabilitation wards. Rehabilitation wards may be more conducive to the practice of team formulation as staff generally know patients over a longer period of time than acute wards. These settings may experience less frequent serious incidents due to lower levels of acute distress, meaning that meetings are less likely to be interrupted and staff could maintain their focus. Nonetheless, the fact that psychologists in the study were frequently omitting key aspects of formulations such as patient strengths, gaols, values and sociocultural factors highlights an area of need for professional development. Formulation is a key competence of psychological practitioners, and the practice of team formulation is growing in popularity to help multidisciplinary teams develop more psychologically‐informed support plans (Association of Clinical Psychologists, 2022). Despite this growing trend, the practice of team formulation is not systematically evaluated on psychological practitioner training programmes, suggesting the need to invest more resource and time in developing competencies in this area (Bucci et al., 2021). The TFQS is a useful tool to aid training and supervision in formulating in teams (Bucci et al., 2021).

The degree to which intervention elements were implemented varied across wards as reflected by the range scores for dosage data. This flexibility and adaptation is a well‐established factor in enabling the successful delivery of complex interventions and psychological interventions on acute wards in particular (Evlat et al., 2021; Moore et al., 2015; Raphael, Price, et al., 2021). The most frequent adaptation was that psychologists focused their time on carrying out therapy with patients and spent less time than planned carrying out work that relied on other staff such as supervising staff to deliver interventions and team formulation. Consistent with previous research (Evlat et al., 2021), interviews and observational we work carried out alongside the trial suggested that management support to ring fence staff time to engage in the intervention is needed to increase staff participation. Consistent with previous research (Evlat et al., 2021), our interviews data also suggested that nurses lacked confidence to deliver the level 2 and that they wanted more opportunities to practice skills alongside psychological therapists.

Limited nurse engagement within the intervention may also reflect the broad nature of the nurse's role (Totman et al., 2011). Nurses are frontline staff and represent a large workforce within the health service. Many ward processes and procedures are therefore typically allocated to nurses, resulting in multiple competing demands (Wyder et al., 2017). Acute mental health wards are risk averse environments where staff blame and accountability are high, the focus of nurse's time and attention therefore easily gets drawn into processes that are mandatory and auditable (Raphael, Hutchinson, et al., 2021). Ward staff reluctance to engage in psychological work and may reflect the rigid nature of professional roles within inpatient settings which prevents effective team working and the delivery of holistic models of care being (Baguley et al., 2007). Despite competing demands on nurse's time and rigid perceptions of professional roles, our study suggests that nurses can ring fence time to engage in psychological work with management support albeit this was extremely patchy across services. Engaging ward managers in the delivery of ward‐based psychological interventions is therefore vital from the outset (Evlat et al., 2021).

Arguably, changing the nurse's role and culture on the ward would also require more time. The intervention period in the study was only 6 months and therefore not long enough to fully embed the model and news ways of working. In support of this argument, meetings which were well established parts of the ward routine such as ward rounds were rarely cancelled despite the occurrence of serious incidents. The prioritization of ward rounds even in the context of serious incidents requiring staff resource also highlights the importance of ensuring that psychological formulations inform discussions during these meetings and that attending ward rounds would be a good use of psychologist's time on the wards.

The TULIPS intervention was delivered by a.5 WTE psychologist per ward. We made the decision to evaluate the impact of a part time role as this was more in keeping with current practice within inpatient wards. However, recent British Psychological Society (2021) guidance on psychological provision for acute care mental health pathways recommends at least one full time psychologist supported by more senior psychologists working across multiple wards and full time assistant psychologist practitioners. It is argued that this critical mass of psychological practitioners is important in shifting the culture of care from a medical to a more psychologically‐informed model of care. In order to maximize the successful implementation of psychological interventions within acute settings and ensure that inpatient environments are more therapeutic, services need to invest in employ a larger psychological workforce in these settings. Ultimately, investing more resource into psychological interventions has the potential to reduce cost by reducing length of staff and the frequency of serious incidents (Berry, Raphael, Haddock, et al., 2022).

The assessment tools (diaries and logs) derived from the fidelity framework were found to be fit for purpose suggested by good completion rates. Most fidelity data came from psychologists' diaries. We regularly reminded the psychologists in supervision sessions of the importance of reliable record keeping. To increase credibility, we also incorporated data from multiple sources and overall there was a good level of concordance between data from different sources. We recommend that other researchers assessing fidelity within studies of psychological interventions in inpatients use our framework and methods to maximize and assess fidelity.

In conclusion, we developed a complex psychological intervention in inpatient mental health settings that could be delivered to high fidelity as it targeted known barriers to implementation. We also devised a comprehensive framework for assessing fidelity that can be replicated by other researchers. Overall, we found that our intervention was delivered to a high standard and was well received by staff and patients but there was relatively low adherence and dosage in areas of the TULIPS which required the participation of the wider multidisciplinary team. Psychologists were able to adapt to this obstacle by focusing on aspects of the intervention over which they had more control such as therapy sessions with patients. However, further time to embed the intervention with ward manager support to ring fence staff to engage in team formulation and deliver level 2 interventions would be optimal in helping to ensure that the whole ward culture is more psychologically‐informed.

## AUTHOR CONTRIBUTIONS

**Katherine Berry:** Conceptualization; funding acquisition; writing – original draft; writing – review and editing; supervision; formal analysis. **Fritz Handerer:** Writing – original draft; formal analysis; supervision; project administration. **Sandra Bucci:** Conceptualization; writing – review and editing; funding acquisition. **Georgia Penn:** Formal analysis; data curation. **Helen Morley:** Supervision; data curation. **Jessica Raphael:** Conceptualization. **Karina Lovell:** Funding acquisition; writing – review and editing. **Owen Price:** Funding acquisition; writing – review and editing. **Dawn Edge:** Funding acquisition; writing – review and editing. **Richard J. Drake:** Funding acquisition; writing – review and editing. **Gillian Haddock:** Conceptualization; funding acquisition; writing – review and editing.

## FUNDING INFORMATION

This project was funded by the National Institute for Health Research (NIHR) Programme Grant for Applied Research RP‐PG‐0216‐20009. This study was supported by the NIHR Manchester Biomedical Research Centre (NIHR 203308). SB discloses support for publication of this work from a National Institute for Health and Care Research research professorship (NIHR300794). The views expressed are those of the authors and not necessarily those of the NIHR or the Department of Health and Social Care.

## CONFLICT OF INTEREST STATEMENT

The authors declare no conflicts of interest.

## ETHICS STATEMENT

Ethical approval was obtained in July 2019 by the Greater Manchester NHS Research Ethics Committee, IRAS ID: 264686.

## CONSENT

All participant will give informed consent to participate in the study.

## Data Availability

Data are available from the lead author upon reasonable request.
